# Potential Biological and Climatic Factors That Influence the Incidence and Persistence of Highly Pathogenic H5N1 Avian Influenza Virus in Egypt

**DOI:** 10.3389/fmicb.2018.00528

**Published:** 2018-03-27

**Authors:** Ahmed H. Salaheldin, Elisa Kasbohm, Heba El-Naggar, Reiner Ulrich, David Scheibner, Marcel Gischke, Mohamed K. Hassan, Abdel-Satar A. Arafa, Wafaa M. Hassan, Hatem S. Abd El-Hamid, Hafez M. Hafez, Jutta Veits, Thomas C. Mettenleiter, Elsayed M. Abdelwhab

**Affiliations:** ^1^Institute of Molecular Virology and Cell Biology, Friedrich-Loeffler-Institut, Federal Research Institute for Animal Health, Greifswald-Insel Riems, Germany; ^2^Institute of Poultry Diseases, Free University of Berlin, Berlin, Germany; ^3^Department of Poultry Diseases, Faculty of Veterinary Medicine, Alexandria University, Edfina, Egypt; ^4^Institute of Mathematics and Computer Science, University of Greifswald, Greifswald, Germany; ^5^Veterinary Serum and Vaccine Research Institute, Cairo, Egypt; ^6^National Laboratory for Veterinary Quality Control on Poultry Production, Animal Health Research Institute, Giza, Egypt; ^7^Faculty of Veterinary Medicine, Damanhur University, Damanhur, Egypt

**Keywords:** H5N1, highly pathogenic avian influenza virus, poultry, meteorological factors, epidemiology, ducks, clade 2.2.1, Egypt

## Abstract

Highly pathogenic H5N1 avian influenza virus (A/H5N1) of clade 2.2.1 is endemic in poultry in Egypt where the highest number of human infections worldwide was reported. During the last 12 years the Egyptian A/H5N1 evolved into several genotypes. In 2007-2014 vaccinated poultry suffered from antigenic drift variants of clade 2.2.1.1 and in 2014/2015 an unprecedented upsurge of A/H5N1 clade 2.2.1.2 occurred in poultry and humans. Factors contributing to the endemicity or re-emergence of A/H5N1 in poultry in Egypt remain unclear. Here, three potential factors were studied: climatic factors (temperature, relative humidity, and wind speed), biological fitness *in vitro*, and pathogenicity in domestic Pekin and Muscovy ducks. Statistical analyses using negative binomial regression models indicated that ambient temperature in winter months influenced the spread of A/H5N1 in different geographic areas analyzed in this study. *In vitro*, at 4 and 56°C 2.2.1.1 and recent 2.2.1.2 viruses were more stable than other viruses used in this study. Further, Pekin ducks were more resistant than Muscovy ducks and the viruses were excreted for up to 2 weeks post-infection assuming a strong role as a reservoir. Taken together, ambient temperature in winter months potentially contributes to increasing outbreaks in some regions in Egypt. Heat stability of clade 2.2.1.1 and recent 2.2.1.2 viruses probably favors their persistence at elevated temperatures. Importantly, asymptomatically infected Pekin ducks may play an important role in the spread of avian and human-like A/H5N1 in Egypt. Therefore, control measures including targeted surveillance and culling of silently infected Pekin ducks should be considered.

## Introduction

Highly pathogenic avian influenza virus (HPAIV) H5N1 (A/H5N1) caused enormous economic losses in poultry in many countries worldwide and genetically diversified into 10 clades and several subclades since 1996/1997 (Smith et al., 2015). Clade 2 viruses spread from China to Europe and Africa since 2003, and eventually became endemic in poultry in Egypt and several Asian countries. Since 2006, Egyptian A/H5N1 of clade 2.2.1 have diversified into several genetic groups. Most of these phylogroups disappeared but two major clades circulated for several years (Abdelwhab et al., 2016). Clade 2.2.1.1 represented antigenic-drift variants, which were primarily isolated from vaccinated commercial poultry leading to three human infections so far according to the official reports to the World Health Organization. These viruses appeared in early 2007 and predominated in 2008-2010 challenging the efficacy of the highly diverse H5 vaccines in Egypt. In 2011-2014, the prevalence of 2.2.1.1 viruses dramatically decreased and they are most likely extinct by now (Abdelwhab et al., 2016; El-Shesheny et al., 2017; Rohaim et al., 2017). The second clade are 2.2.1.2 viruses which circulated in non-vaccinated backyard birds and were introduced into small-scale farmed poultry since early 2008. The vast majority of infected humans were infected by this genotype (Younan et al., 2013). In 2014/2015, an unprecedented upsurge of 2.2.1.2 was reported in poultry and humans marking Egypt as the country with the highest number of human infections with A/H5N1 worldwide (Arafa et al., 2015; WHO, 2017). These viruses spread to neighboring countries posing a serious threat in the Middle East (Naguib et al., 2016a; Salaheldin et al., 2017). Driving forces for the emergence, extinction or spread of A/H5N1 clades in Egypt are not well-studied except for the massive application of vaccines and antivirals in poultry (Abdel-Moneim et al., 2011; Cattoli et al., 2011; Abdelwhab et al., 2016; El-Shesheny et al., 2016; Naguib et al., 2016b).

The spread of influenza viruses may be influenced by several factors related to environment, virus, and host. Previous research has shown that meteorological factors, biological fitness, and/or domestic ducks play significant roles in shaping the spread of influenza viruses (Li et al., 2004, 2015). Apart from the seasonal incidence of human influenza viruses, there is a paucity of information about the impact of climatic factors on the spread and course of influenza viruses' infection in domestic birds. Studies on wild bird populations showed that the regional prevalence of avian influenza viruses (AIV) may follow a seasonal pattern and can be influenced by climatic conditions (Gilbert et al., 2008a; Herrick et al., 2013; Ferenczi et al., 2016). However, little is known about the correlation and the impact of climatic factors on introduction and persistence of HPAIV in domestic poultry. Biological fitness (e.g., stability in harsh niches, rapid replication, or spread) may be advantageous for virus perpetuation outside the host or increase adaptation to birds and human (Terregino et al., 2009). The role of domestic ducks for generation and persistence of A/H5N1 in Asian countries is well-studied. In contrast to the high mortality in chickens, domestic ducks were considered a “Trojan horse” because they are usually infected without exhibiting clinical signs or mortality (Chen et al., 2004; Hulse-Post et al., 2005; Gilbert et al., 2006; Songserm et al., 2006) enabling silent spread of the virus to other hosts (e.g., chickens, humans) (Kim et al., 2009; Lebarbenchon et al., 2010). In Egypt, about 40 million ducks raised in backyard and commercial farms where Pekin and Muscovy ducks are the most prevalent breeds (Hassan et al., 2013). A/H5N1 was isolated from asymptomatic domestic ducks in hot summer seasons in some localities in Egypt (Hassan et al., 2013). Also, viruses isolated from different organs of ducks were more genetically diverse than those isolated from chickens (Watanabe et al., 2011a), suggesting a role for ducks in perpetuating the endemicity of A/H5N1 in Egypt.

Here, we investigated three potential factors which could affect the evolution of A/H5N1 in poultry in Egypt: the climatic factors from October to March where the incidence of outbreaks rises, the variation in biological fitness of 2.2.1.1 and 2.2.1.2 viruses in different cells, and the role of Pekin and Muscovy ducks as a reservoir for two representative viruses from both major clades isolated from poultry in Egypt.

## Materials and methods

### Climatic and epidemiological data collection and processing

**Figure 1:**
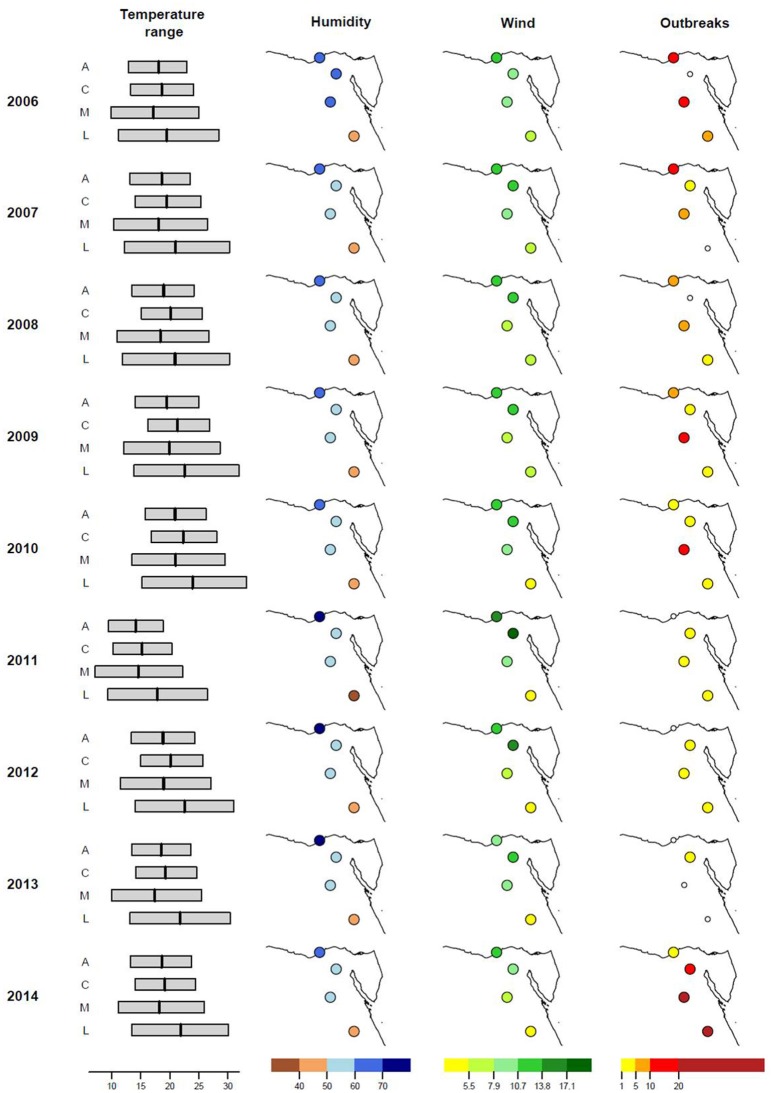
Climatic factors and number of H5N1 outbreaks in four selected governorates in Egypt. Illustrated are the ranges from minimum to maximum temperature (°C, average temperature marked as vertical line), relative humidity (%), wind speed (m/s), and number of avian influenza outbreaks in domestic poultry for four selected provinces in Egypt (from north to south: A, Alexandria; C, Cairo; M, Minya; L, Luxor) from 2006 to 2015 (October 1st to March 31st of the next year).

The daily data on temperature (minimum, maximum, and average), relative humidity, and wind speed were retrieved from Weather Underground Website (Dugas et al., 2013). Data were collected for 10 seasons from 2006 to 2015. Each season lasted from the 1st of October to 31st of March of the following year, when the incidence of outbreaks peaked according to previous surveillance (Arafa A. et al., 2012; Arafa A. S. et al., 2012; El-Zoghby et al., 2013; Arafa et al., 2015; Kayali et al., 2016). For example, the “2006-2007” season represented the data from 01.10.2006 to 31.03.2007. The means and standard deviations for each month were calculated and used for analysis. Four governorates namely Alexandria, Cairo, Minya, and Luxor (ordered from north to south) were selected based on their geographic location, climate zones, and variable number of A/H5N1 outbreaks (Figure 1). While Alexandria is situated close to the Mediterranean coast and therefore characterized by a maritime climate with higher precipitation and moderate temperatures, following the river Nile further south the climate changes into a hot desert climate with little to no precipitation and high temperatures. The total number of A/H5N1 outbreaks per month was summarized for each season based on the official reports of national surveillance conducted by the Egyptian National Laboratory for Veterinary Quality Control on Poultry Production (NLQP) and reported to the World Organization for Animal Health (OIE) and to Food and Agriculture Organization of the United Nations (FAO). Five unreported cases, which were detected in a retrospective surveillance from vaccinated birds, were also included in the analysis. The effect of selected climate parameters on the number of outbreaks in each region and all-over Egypt was analyzed as described below.

The correlation between each of the climate factors and the reported number of outbreaks was calculated as Spearman's rank correlation coefficient for the whole of Egypt and also for each of the governorates. Furthermore, negative binomial regression models were used to assess the combined contribution of several climate factors on the observed number of outbreaks per governorate. We investigated a “full” model, which includes regional effects and all climate factors as explanatory variables. Since climate factors are interrelated, significant effects of these variables might be obscured in the full model. Thus, a “reduced” model with fewer variables was derived from the full model by stepwise backward elimination minimizing the Akaike information criterion (AIC). AIC was chosen for model selection for the reason that this criterion evaluates the goodness of fit (based on the likelihood) and simultaneously takes the number of explanatory variables into account. Additionally, we investigated a model based solely on the available climate variables without correcting for regional differences and an additional model which includes only regional effects and hence assumes that the observed numbers of outbreaks follow a random (climate independent) pattern.

### Viruses and cells

To study the biological fitness of different A/H5N1 clades in Egypt (2.2.1, 2.2.1.1, and 2.2.1.2), six viruses were obtained from the repository of the Friedrich-Loeffler-Institut (FLI), Germany as summarized in Table 1. Two viruses belonged to the clade 2.2.1, A/chicken/Egypt/083-NLQP/2008(H5N1) (designated 2.2.1-A), A/chicken/Egypt/0815-NLQP/2008(H5N1) (designated 2.2.1-B), one virus belonged to the early clade 2.2.1.2 (A/duck/Egypt/0897-NLQP/2008(H5N1) (designated 2.2.1.2-A), one virus of clade 2.2.1.2 originate from the recent upsurge in 2014/2015 A/turkey/Egypt/AR238-SD177NLQP/2014(H5N1) (designated 2.2.1.2-C) and a putative predecessor virus from 2013 A/chicken/Egypt/NLQP7FL-AR747/2013(H5N1) (designated 2.2.1.2-B). The last virus belonged to clade 2.2.1.1 A/chicken/Egypt/0879-NLQP/2008(H5N1) (designated 2.2.1.1) which was extensively studied as an A/H5N1 immune-escape variant in vaccinated chickens (Abdelwhab et al., 2011; Grund et al., 2011). It is antigenically distinct from clade 2.2.1.2 viruses. Furthermore, in addition to 2.2.1.1 virus, A/turkey/Egypt/R1507/2016 (designated 2.2.1.2-D) isolated in 2016 from a vaccinated turkey flock (Salaheldin et al., 2017) was used for infection of Pekin and Muscovy ducks. The 2.2.1.2-D virus was not available when the project started. Not all *in-vitro* characterization experiments were done for this virus and therefore these data (except for receptor binding) are not provided. The 2.2.1.2-D was used for the infection of ducks because it was the most recent Egyptian isolate from clade 2.2.1.2. Lastly, A/PR/8/1934(H1N1) (designated PR8), a human virus, and A/quail/California/D113023808/2012(H4N2) were used as controls in receptor binding assays. The H4N2 virus was kindly provided by Beate Crossley, UC Davis. Viruses were inoculated into the allantoic cavity of specific pathogen free (SPF) embryonated chicken eggs (ECE) for 3–5 days. Chorioallantoic fluid (AF) was tested by hemagglutination test using 1% chicken erythrocytes (OIE, 2015). Bacteria-free AF was pooled and virus was titrated by plaque assay as described below.

**Table 1:** Viruses isolated or used in this study.

**H5N1 Viruses**	**Abbreviation**	HA accession number^*^	**Clade**
**VIRUSES USED IN BIOLOGICAL CHARACTERIZATION**			
A/chicken/Egypt/083-NLQP/2008	2.2.1-A	CY044032	2.2.1
A/chicken/Egypt/0815-NLQP/2008	2.2.1-B	GQ184221	2.2.1
A/chicken/Egypt/0879-NLQP/2008	2.2.1.1	GQ184238	2.2.1.1
A/duck/Egypt/0897-NLQP/2008	2.2.1.2-A	JF746738	2.2.1.2^**^
A/chicken/Egypt/NLQP7FL-AR747/2013	2.2.1.2-B	EPI557170	2.2.1.2
A/turkey/Egypt/AR238-SD177NLQP/2014	2.2.1.2-C	EPI573268	2.2.1.2
**VIRUSES USED IN DUCK EXPERIMENT**			
A/chicken/Egypt/0879-NLQP/2008	2.2.1.1	GQ184238	2.2.1.1
A/turkey/Egypt/AR1507/2016	2.2.1.2-D	EPI827065	2.2.1.2

* *GISAID/GenBank accession numbers*,

** *Early 2.2.1.2 = 2.2.1/C clade*.

Madin-Darby canine kidney cells II (MDCKII) and adenocarcinomic human alveolar basal epithelial cells (A549) were obtained from the FLI. Chicken embryo kidney cells (CEK) were prepared from kidneys of 18-day-old SPF ECE (Lohmann Animal Health, Germany) according to Standard procedures.

### Plaque assay

Titration of viruses was performed in MDCKII by ten-fold serial dilutions. The cells were infected for 1 h, then washed twice with PBS and overlaid with 3 ml plaque test medium, minimal essential medium (MEM) containing 37% fetal calf serum (Sigma, Germany) and 1.8% Bacto-agar (BD, USA) at a 1:1 ratio. Trypsin was added not to the cells because HPAIV can grow in the presence of serum without trypsin. Plates were incubated at 37°C, 5% CO_2_ for 3 days and then fixed with formalin containing crystal violet. The number of plaques was counted and the final titers were calculated and expressed as plaque forming unit per ml (PFU/ml). For measuring the size of plaques produced by different viruses in MDCKII, Nikon Instruments NIS elements basic research software was used.

### Replication kinetics

CEK, MDCKII and A549 cells were infected with 1PFU per 1000 cells in 2–4 independent assays. After 1 h, cells were washed with citrate-buffered saline (CBS) pH 3.0 to inactivate extracellular virions. Then, the cells were washed twice with isotonic PBS, and infection medium, MEM with bovine serum albumin (BSA), was added and incubated for 1, 8, 24, 48, and 72 h post-infection (hpi) at 37°C and 5% CO_2_. Harvested cells and supernatants were stored at −80°C until use. The results were expressed as average and standard deviation of PFU/ml of all replicates.

### Thermo- and pH stability

The titre of indicated viruses used in biological characterization (Table 1) were adjusted to 10^5^-10^6^ PFU/ml and aliquots were incubated in duplicates at 4°C for 1, 2, 3, and 4 months. Also, duplicates were incubated at 56°C for 0, 1, 2, 3, and 4 h or were incubated with an equal volume with PBS pH 4, 5, 6, 7, or 7.4 at room temperature (20 to 22°C) for up-to 7 days followed by neutralization with sodium hydroxide. Aliquots were removed and stored at −80°C until use. The decrease in HA titre and infectivity was investigated using HA test and plaque assay, respectively. The HA test was conducted in duplicate for each replicate. The experiments were repeated twice and the average and standard deviation of each experiment were given.

### Receptor binding assay

Affinity to avian α2,3 and human-like α2,6- sialic acid (SA) receptors was assessed using modified turkey erythrocytes (TRBCs) (Herfst et al., 2012). Briefly, SA was removed from TRBCs by incubation with Vibrio cholerae neuraminidase (Sigma-Aldrich, Germany) in the presence of calcium chloride (Herfst et al., 2012). After washing with PBS, desialylated TRBCs were suspended in PBS containing 1% BSA. Complete loss of hemagglutination of the TRBCs was confirmed by incubation with control viruses (i.e., PR8 and H4N2). Resialylation was done using α2,6-(N)-sialyltransferase (Takara, Germany) or α2,3-(N)-sialyltransferase (Sigma-Aldrich, Germany) in final concentrations of 1.5 mM Cytidine 5′-monophosphate (CMP)-sialic acid (Sigma-Aldrich, Germany). Modified TRBCs were suspended in PBS containing 1% bovine serum albumin to a final concentration of 0.5%. Resialylation was confirmed by hemagglutination of viruses using human PR8 with high affinity to α2,6-SA and H4N2 with high affinity to avian α2,3-SA receptors. HA test was done using the modified TRBCs, desialylated RBCs and original turkey RBCs (OIE, 2015). The assay was run in duplicates and repeated twice.

### Experimental infection of ducks

The animal experiment in this study was conducted in the biosafety level 3 animal facilities of the FLI following the German Regulations for Animal Welfare after approval by the authorized ethics committee of the State Office of Agriculture, Food Safety, and Fishery in Mecklenburg—Western Pomerania. The experiment was approved by the commissioner for animal welfare at the FLI representing the Institutional Animal Care and Use Committee.

Sixty 4 to 5-week-old Pekin (*n* = 30) and Muscovy (*n* = 30) ducks were purchased from a commercial, influenza-free breeder flock. Birds were housed for 4 days before virus inoculation. Water and feed were supplied *ad-libitum*. Blood samples as well as oropharyngeal and cloacal swabs were collected pre-infection. At day of inoculation (day 0), 10 birds per group were inoculated via the oculo-nasal route with 0.2 ml inoculum containing 10^5^ PFU/bird of each virus. At day 1 post-inoculation (dpi), 5 sentinel birds were added to each group. Ducks were observed daily for 14 days post-inoculation. Pathogenicity index (PI) based on clinical scoring was done as following: 0 for healthy birds, 1 for birds with one clinical sign (depression, nervous signs, respiratory signs, diarrhea, or facial oedema), 2 for birds that showed more than one clinical sign, and 3 for dead birds. Moribund birds which could not eat or drink were euthanized and scored 3 at the next day. The PI was calculated from the daily mean score of all birds during a 14-day observation period. All survived birds at the end of the experiment were anesthetized by inhalation of isoflurane (CP-Pharma, Germany), and then slaughtered and the blood was collected from the jugular veins.

To determine the level of viral excretion swabs were collected in MEM containing BSA and antibiotics from surviving ducks at 2, 4, 7, 11, and 14 dpi. Also, lung and spleen samples were collected at 3 dpi from three birds killed for histopathology as described below. Viral RNA was extracted from swabs using NucleoSpin 8/96 PCR Clean-up Core Kit (Macherey & Nagel GmbH) and from organs using NucleoMag kit according to the manufacturer instructions in automatic extraction. Real-time reverse-transcription polymerase chain reaction (RT-qPCR) targeting the matrix gene was used (Hoffmann et al., 2016). Standard curves for virus quantification were generated in each RT-qPCR plate using RNA extracted from 10-fold serial dilutions of 2.2.1.1 virus. Ct values of samples were plotted against the standard curves and the results were presented as PFU/ml.

To investigate the distribution of influenza antigens in different tissues, 3 birds per group were euthanized at 3 dpi by isoflurane inhalation and blood withdrawal. Samples were collected from trachea, lung, heart, spleen, liver, pancreas, duodenum, jejunum, cecal tonsils, bursa of Fabricius, thymus, and brains. All samples were fixed in formalin and embedded in paraffin-wax, then subjected to histopathologic and immunohistochemistry (IHC) examination. Primary anti-influenza NP-antibodies and secondary biotinylated goat anti-rabbit IgG1 (Vector) antibodies (1:200) were used to detect H5N1 antigens in different tissues (Klopfleisch et al., 2006). The intensity of signals of influenza nucleoprotein was semi-quantified by scoring on a 0 to 4 scale for tissues: 0 = negative; 1 = single cells, 2 = scattered foci, 3 = numerous foci, 4 = coalescing foci or diffuse; and on a scale of 0 to 3 for endothelium: 0 = negative; 1 = single blood vessel, 2 = multiple blood vessels, 3 = diffuse, as described previously (Klopfleisch et al., 2006).

Serum samples collected at day 0 and at the end of the experiment from surviving birds were inactivated at 56°C for 2 h and tested by a commercial enzyme linked immunosorbent assay (ELISA) targeting the NP of AIV as recommended by the manufacturer (ID Screen® Influenza A Antibody Competition Multi-species, IDvet). The results of ELISA were confirmed using hemagglutination inhibition (HI) test against 8 HAU of the challenge viruses (OIE, 2015).

### Statistic analysis

For the duck experiment, differences in viral excretion at 2 and 4 dpi, respectively, were evaluated using Kruskal-Wallis tests followed by Wilcoxon tests using the Benjamini-Hochberg procedure for multiple testing correction. Clinical scoring was compared across groups based on the mean clinical score per bird during a 14 days observation period in the same manner. *In vitro* replication kinetics were analyzed by a repeated measures ANOVA with Benjamini-Hochberg-corrected *post-hoc* tests. All computations in this study were performed in R version 3.3.1 from the R-project website (http://www.r-project.org) with packages nlme, car, multcomp and MASS (Venables and Ripley, 2002; Hothorn et al., 2008; R Core-Team, 2015; Pinheiro et al., 2017).

## Results

### Ambient temperature influenced the prevalence of A/H5N1 outbreaks in examined regions in Egypt

Data on temperature, humidity, and wind speed from 2006 to 2015 were analyzed for overall Egypt and for four selected provinces (Figure 1). In 2006, 185 outbreaks of avian influenza in domestic poultry were reported in Egypt. In the following 2 years, the number of outbreaks decreased to 69 cases in 2008 but increased to over 200 cases in 2009 and 2010. After a decline for the following 3 years, the number of outbreaks peaked at 445 cases in 2014/2015, where Cairo, Minya, and Luxor had the highest number of outbreaks. On the contrary, Alexandria showed a steady decrease in outbreaks from 2006 onwards.

Potential correlations of single climate factors with outbreaks of A/H5N1 in Egypt were analyzed by Spearman's correlation coefficients (ρ). As shown in Table 2, only weak to moderate correlations were observed for overall Egypt and also for each of the governorates. For instances, the decrease in the number of outbreaks in Alexandria correlates with an increase of humidity (ρ = −0.61) and the increase of outbreaks in Minya correlated with an increase of the minimum temperature per winter season (ρ = 0.45). However, none of the correlations were statistically significant (*P* < 0.05). Accordingly, the observed variation in A/H5N1 outbreaks could not be attributed to a single climate factor.

**Table 2:** Correlation of climatic factors with A/H5N1 outbreaks in poultry in all Egypt and the four selected governorates.

	**Temp. (min)**	**Temp. (max)**	**Temp. (average)**	**Humidity (%)**	**Wind speed**
Entire Egypt	0.38	0.03	0.35	−0.18	0.29
Alexandria	−0.03	−0.03	0.17	−0.61	0.39
Cairo	0.28	0.23	0.20	0.02	−0.02
Minya	0.45	0.29	0.37	0.17	−0.14
Luxor	0.21	−0.16	0.19	0.17	−0.10

*Spearman's correlation coefficient*.

*None of the reported correlations is statistically significant (P < 0.05)*.

Nevertheless, the variation in A/H5N1 outbreaks might have been influenced by a combination of several climate factors. Therefore, five negative binomial regression models, which differed in the choice of explanatory variables (with/without climate factors and with/without regional effects, one model resulted from the variable selection procedure), were constructed for the governorate-level data. All proposed negative binomial regression models showed similar deviances and fit reasonably well to the data according to goodness of fit tests (χ^2^ tests on the deviances) (Table 3). However, likelihood ratio tests comparing the full model with all other proposed models indicated that incorporation of both regional and climate effects significantly improved model performance. Yet some climate variables may be omitted from the full model as the difference in log-likelihood between our reduced model (resulting from the variable selection procedure) and the full model was not statistically significant. As expected, our reduced model showed the best fit regarding the AIC. It comprised regional effects, maximum temperature and average temperature as explanatory variables. The regression coefficients indicated a significantly increased baseline risk for Minya and Luxor, but not for Cairo, in comparison to Alexandria (Table 3; Supplementary Figure S1). Furthermore, according to this model an increase in maximum temperature (while average temperature remains unchanged) corresponded to a decrease in the number of outbreaks (β = −2.38). Conversely, an increase in average temperature (while maximum temperature remains unchanged) corresponded to an increase in the number of outbreaks (β = 2.79). Both effects were statistically significant (Table 3). Thus, an increase in average temperature appears to promote A/H5N1 outbreaks whereas very hot days in winter seasons presumably counteract this trend.

**Table 3:** Negative binomial regression models for explaining the observed number of A/H5N1 outbreaks in winter season (d.f., degrees of freedom).

	**Model parameters**				**Model performance**				
		**β**	**SE**	***P*-value (Wald test)**	**Deviance**	***P*-value (χ^2^ test, goodness of fit)**	**AIC**	**2 x log-likelihood**	***P*-value (Likelihood ratio test to full model)**
Full model	Cairo	−1.45	1.25	0.24	39.78	0.05	206.16	−186.16	–
	Minya	6.38	3.00	0.03	(27 d.f.)				
	Luxor	4.68	4.10	0.25					
	Temp (min)	0.46	1.40	0.75					
	Temp (max)	−2.02	1.08	0.06					
	Temp (average)	1.92	2.30	0.40					
	Humidity	−0.08	0.08	0.33					
	Wind	−0.09	0.17	0.59					
Reduced model	Cairo	−0.32	0.60	0.59	39.82	0.11	201.00	−187.00	0.84
	Minya	7.53	2.27	<0.01	(30 d.f.)				
	Luxor	6.64	2.53	<0.01					
	Temp (max)	−2.38	0.79	<0.01					
	Temp (average)	2.79	0.88	<0.01					
Climate factors only	Temp (min)	0.39	0.98	0.69	39.41	0.12	211.14	−197.14	0.01
	Temp (max)	0.12	0.74	0.87	(30 d.f.)				
	Temp (average)	−0.51	1.69	0.76					
	Humidity	0.09	0.04	0.03					
	Wind	−0.29	0.16	0.06					
Regional effects only	Cairo	−0.65	0.65	0.31	39.74	0.16	208.28	−198.28	0.03
	Minya	0.86	0.63	0.17	(32 d.f.)				
	Luxor	−0.25	0.64	0.70					
Null model					39.92	0.26	208.26	−204.26	0.02
					(35 d.f.)				

*Underlined values indicate significant difference (P < 0.05)*.

This finding could be validated using the nation-level data for all Egypt. Based on this data, a negative binomial regression model using only average temperature and maximum temperature per winter season as explanatory variables indicated that an increase in average temperature increased the risk of an outbreak (β = 1.79) while an increase in maximum temperature decreased the risk of an outbreak (β = −1.50). Again, both effects were statistically significant (Supplementary Table S1, Supplementary Figure S2). Hence, even though this model represents a strong simplification of the actual outbreak scenario, it reveals a potential connection between ambient temperature and A/H5N1 outbreaks and confirms the findings of the best fitting-model for the governorate-level data.

Taken together, ambient temperature is a potential driving climate factor for A/H5N1 outbreaks in Egypt. Our results imply that average temperature of the respective winter season affected the number of outbreaks. Since all models simplify the complexity of the epidemiology of A/H5N1 in Egypt, none of them fully explains the observed variation in A/H5N1 outbreaks (Supplementary Figure S1, Supplementary Figure S2).

### Viruses in clade 2.2.1.1 and recent 2.2.1.2 exhibit increased stability at low and high temperature

**Figure 2:**
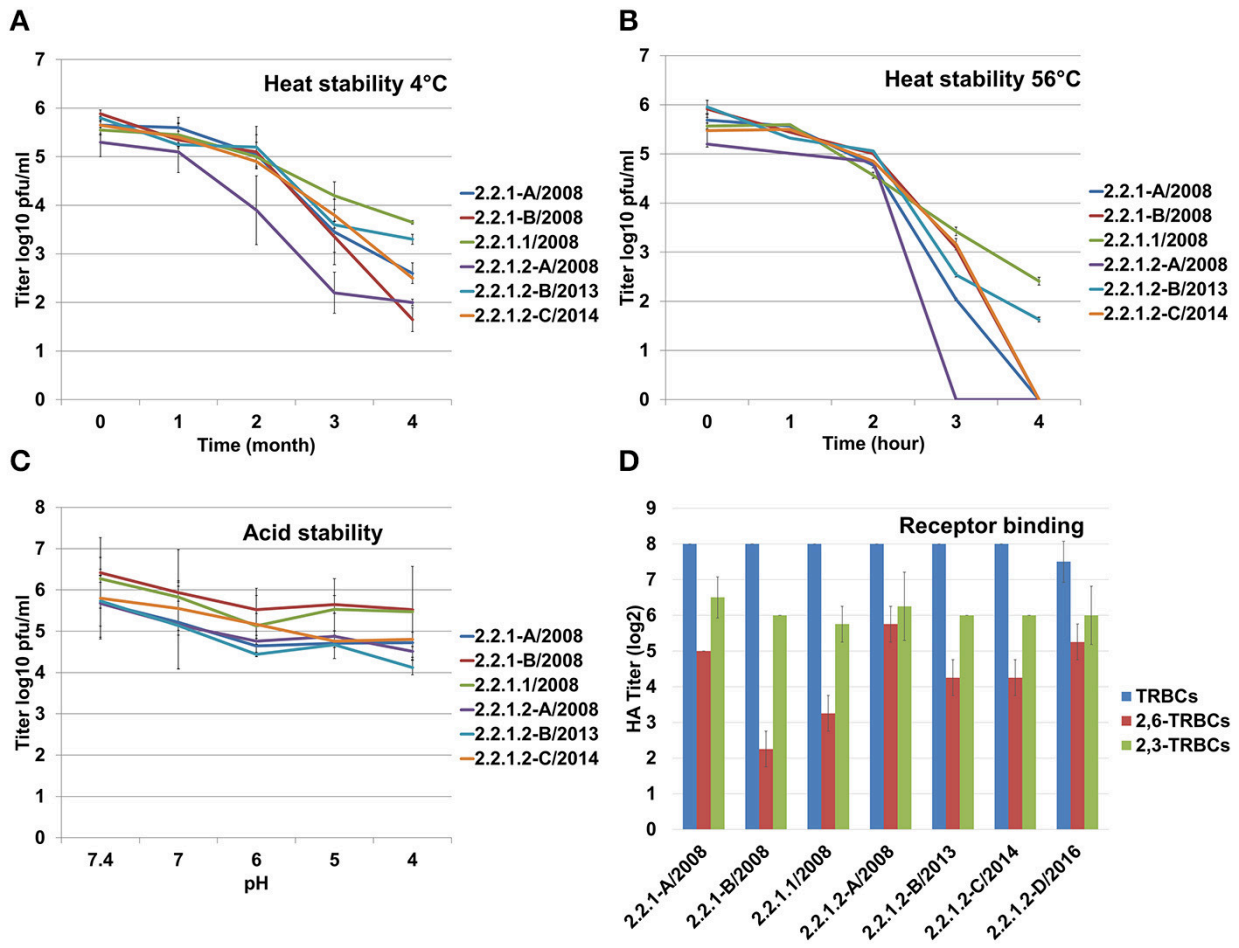
Stability of the Egyptian H5N1 viruses at different temperatures and pHs. Heat stability at 4°C **(A)** or 56°C **(B)**, and acid stability **(C)**. **(D)** Receptor binding affinity was tested against turkey RBCs (TRBCs) and modified TRBCs carrying avian α2,3 and human-like α2,6- sialic acid. All experiments were conducted in duplicates, heat stability at 56°C and acid stability was assessed in two independent experiments. Shown are the average and standard deviations of all experiments. Titration of viruses was carried out in MDCKII cells.

At 4°C, all viruses survived for at least 4 months. The 2.2.1.1 virus and 2.2.1.2-B were more stable and showed the highest titres compared to the other viruses used in this study (Figure 2A). After 4 months, 2.2.1.1 virus and 2.2.1.2-B had 10- to 100-fold higher titres than the other viruses (Figure 2A). Likewise, at 56°C all viruses were relatively stable for 2 h. The recent 2.2.1.2 and 2.2.1.1 viruses were more stable than other viruses after 3 h. The 2.2.1.1 virus and 2.2.1.2-B were not totally inactivated even after 4 h (Figure 2B). Moreover, all viruses were stable at different pH for 7 days. 2.2.1.2-B showed a ~10-fold decreased titre at lower pH (Figure 2C).

### Antigenic drift 2.2.1.1 virus replicated at lower levels in human lung cells compared to the other viruses

**Figure 3:**
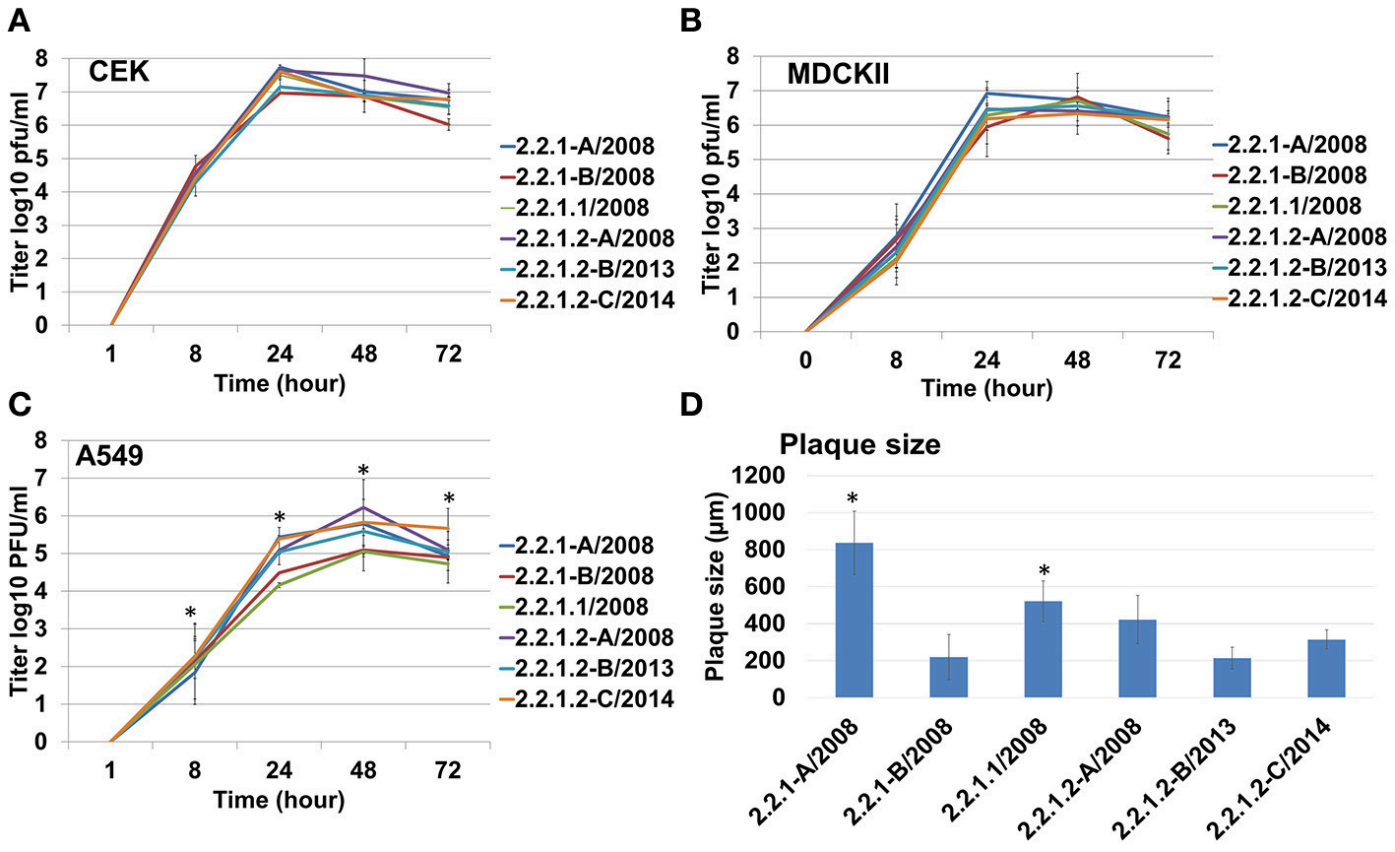
Replication and cell-to-cell spread of different Egyptian-origin H5N1 viruses. Replication of different viruses in chicken embryo kidney (CEK) cells **(A)**, Madin-Darby canine kidney type II (MDCKII) cells **(B)**, human adenocarcinoma lung cells (A549) **(C)**. All experiments were repeated 2–4 times. Cell-to-cell spread was analyzed by measuring plaque size induced by different viruses in MDCKII cells **(D)**. ^*^P < 0.05.

All Egyptian viruses reacted at similar levels using unmodified TRBCs (HA titre ~256) as well as against α2,3-SA carrying RBCs (HA titre 64 to 128). The viruses varied in binding to α2,6-SA RBCs although at 2- to 16-fold lower efficiency than to avian α2,3-SA receptors. The 2.2.1.1 and 2.2.1-B reacted 2- to 8-fold less than other viruses. 2.2.1.2-D bound to α2,6-SA receptors 4-fold higher than 2.2.1.1 (Figure 2D). PR8 bound at similar levels to unmodified TRBCs and α2,6-SA carrying RBCs and did not bind to the avian α2,3-SA receptors. Conversely, H4N2 did not bind to α2,6-SA-TRBCs (data not shown). All viruses reached the maximum titer at 24 hpi in CEK (Figure 3A) and MDCKII (Figure 3B). Interestingly, in A549, 2.2.1.1 replicated at significantly lower titre than 2.2.1.2-C at 8, 24, 48, and 72 hpi and lower than 2.2.1-A at 24 hpi (Figure 3C). While 2.2.1-A produced the largest plaques, 2.2.1-B and 2.2.1.2-B produced the smallest plaques. Plaque size induced by the 2.2.1.1 virus was significantly larger than the recent 2.2.1.2 viruses (2.2.1.2-B and 2.2.1.2-C) (Figure 3D).

### Pekin ducks are more resistant than Muscovy ducks toward infection with different egyptian H5N1 viruses

**Figure 4:**
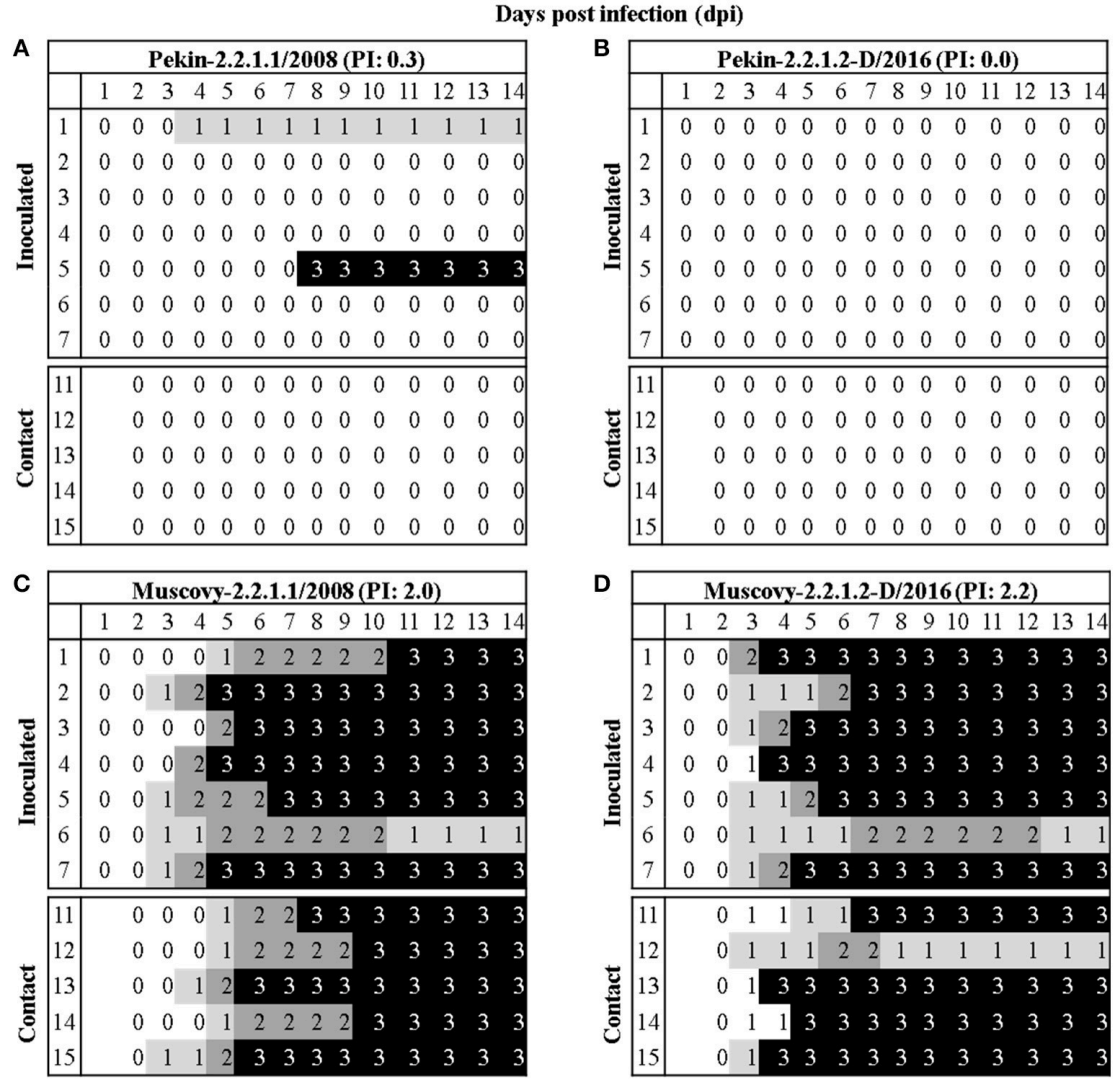
Morbidity and mortality in Pekin and Muscovy ducks after inoculation with two different A/H5N1 of clades 2.2.1.1 and 2.2.1.2. Clinical scoring after oculonasal infection of 4–5 weeks old Pekin **(A,B)** or Muscovy **(C,D)** ducks with H5N1 viruses belonging to clades 2.2.1.1 **(A,C)** or 2.2.1.2 **(B,D)**. At 1 day post inoculation, five naïve ducks were added to assess virus transmissibility. All birds were observed for up to 14 days. Ducks without clinical symptoms were scored “0”. The score “1” was applied to ducks showed one of the following clinical signs: depression, ruffled feathers, diarrhea, discharges, torticollis, opisthotonus, or rolling. These ducks were categorized as ill. Severely ill ducks showed two or more clinical signs were scored “2,” whereas dead ducks were scored “3.” Birds which could not eat or drink were euthanized and scored 3 at the next day of observation. The pathogenicity index (PI) for each group was expressed as the mean sum of the daily arithmetic mean values divided by 14; the number of observation days.

Pekin ducks (groups 1 and 2) were more resistant than Muscovy ducks (groups 3 and 4) after inoculation with the Egyptian H5N1 viruses of clade 2.2.1.1 and 2.2.1.2 (Figure 4). A total of 1/7, 0/7, 6/7, and 6/7 inoculated birds died in groups 1 to 4, respectively. None of contact Pekin ducks died, whereas 5/5 and 4/5 contact Muscovy ducks died in groups 3 and 4, respectively (Figure 4). In group 1, all inoculated Pekin ducks except bird number 1 and 5 were clinically healthy up to the end of the experiment. Bird number 1 showed depression beginning at 4 dpi, whereas bird number 5 showed severe nervous signs and therefore was killed at 7 dpi and scored dead at day 8 post-inoculation (Figure 4A). In group 2, all inoculated Pekin ducks remained healthy. Likewise, all contact ducks in both groups were apparently healthy (Figure 4B). On the contrary to Pekin ducks, the majority of Muscovy ducks inoculated with 2.2.1.1 (group 3) or 2.2.1.2 (group 4) died by day 11 post-inoculation. Clinical signs started at 3 dpi with no significant difference in groups 3 and 4 (*p* = 0.29). In group 3, 6 out of 7 Muscovy birds died with a mean death time of 6.5 days and a PI of 2. All contact birds in this group died by day 10 post-inoculation after showing mild to severe clinical signs. Clinical signs started with depression and progressed quickly to become moderate to severe (Figure 4C). In group 4, 6 out of 7 inoculated birds died by day 7 post-inoculation with a mean death time of 5.2 days and a PI of 2.2. The onset of death in group 4 started 1 day earlier than in group 3. Four out of 5 contact birds died in group 4 by day 7. The onset of death started at 4 dpi, 2 days earlier than in group 3 (Figure 4D). One inoculated bird each from groups 3 and 4, and 1 contact bird from group 4 survived. However, they remained sick during the duration of the experiment showing torticollis but were able to obtain food and water.

**Figure 5:**
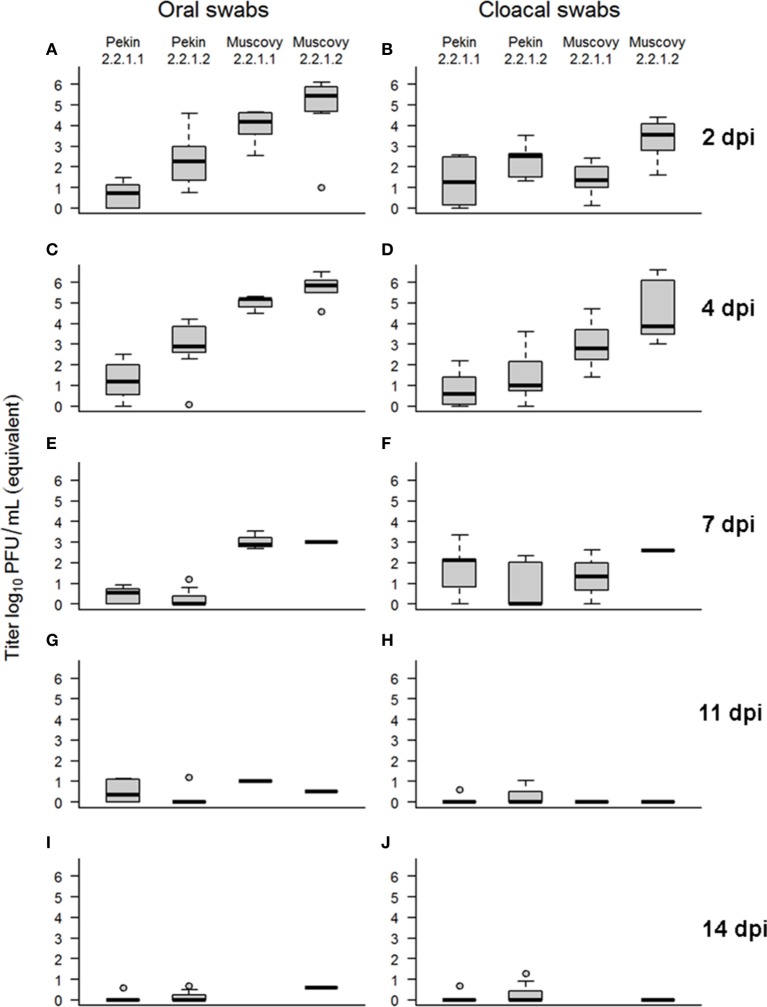
Virus excretion in swab samples collected from Pekin and Muscovy ducks at different time points after infection with Egyptian 2.2.1.1 or 2.2.1.2 viruses. Swab samples were collected from surviving birds at 2 **(A,B)**, 4 **(C,D)**, 7 **(E,F)**, 11 **(G,H)**, and 14 **(I,J)** days post inoculation (dpi) and examined by real-time reverse transcription polymerase chain reaction. Standard curves were generated using serial dilutions of 2.2.1.1 virus. Amount of virus excretion was determined by plotting CT against the log of 2.2.1.1 dilution expressed in equivalent log10 PFU/ml.

At 2 dpi, the majority of ducks in group 1 and all ducks in groups 2, 3, and 4 excreted viruses confirmed by oral and cloacal swabs (Figure 5, Supplementary Table S2). Muscovy ducks excreted significantly higher amounts of the viruses than Pekin ducks. Also, the level of virus excretion in ducks inoculated with the 2.2.1.2 virus at 2 dpi was significantly higher than those infected by 2.2.1.1 virus. At day 4, the majority of Pekin ducks excreted viruses although at lower levels than Muscovy ducks. All Muscovy ducks excreted viruses orally and/or cloacally. Muscovy ducks infected with 2.2.1.2 had a higher amount of virus in cloacal swabs than those infected with 2.2.1.1 (*p* = 0.0078). At 11 and 14 dpi some surviving ducks secreted viruses orally and/or cloacally, although at low levels (Supplementary Table S2, Figure 5).

**Figure 6:**
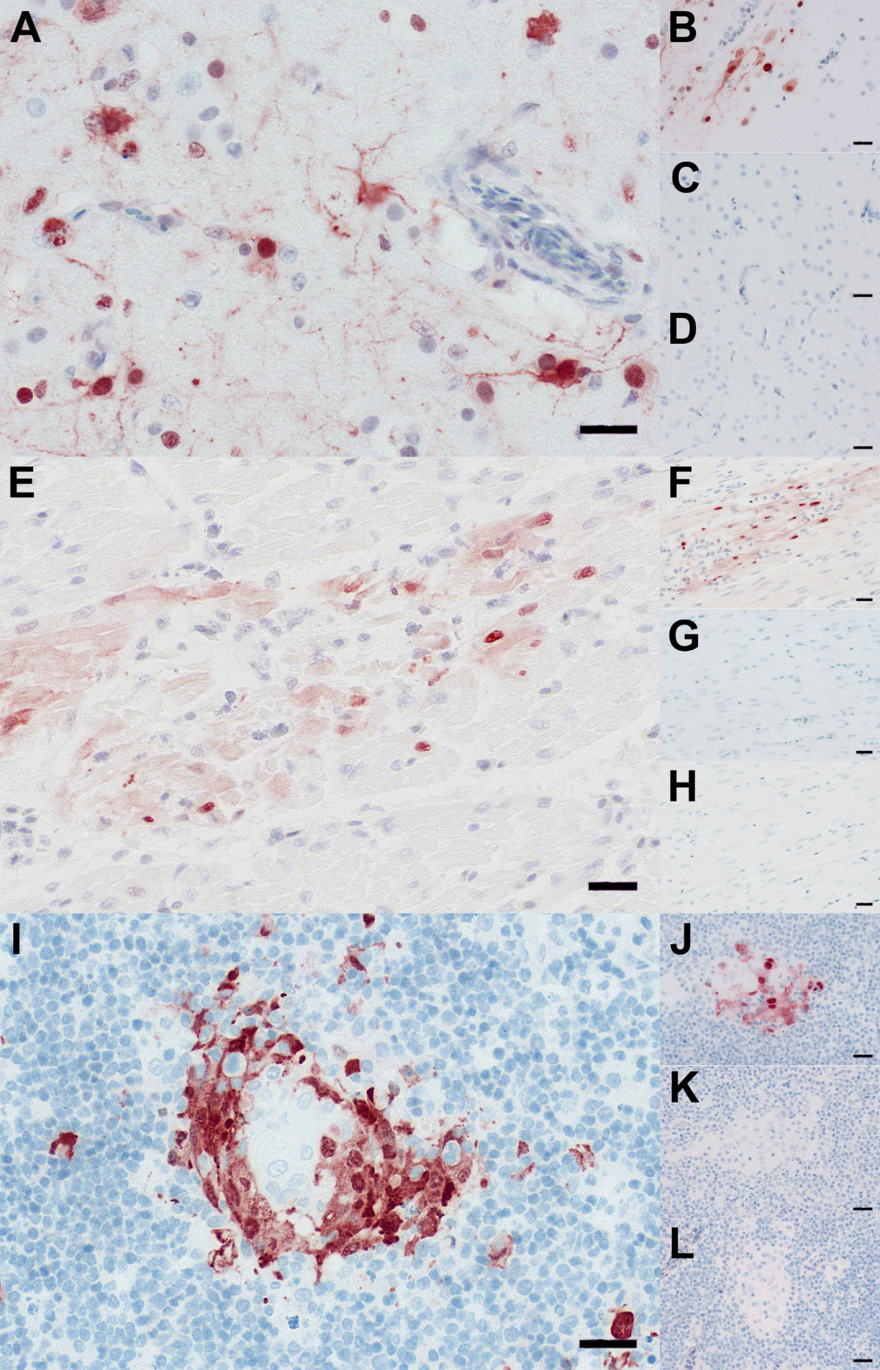
Distribution of A/H5N1 in tissues of Pekin and Muscovy ducks. Immunohistochemistry revealed influenza A-virus nucleoprotein-antigen in neuroglial cells within the brain **(A,B)**, cardiomyocytes within the heart **(E,F)**, and thymocytes within the thymus **(I,J)** of Muscovy ducks **(A,B,E,F,I,J)**. In contrast, influenza A-virus nucleoprotein-antigen was not detected within brain **(C,D)**, heart **(G,H)**, and thymus **(K,L)** of Peking ducks **(C,D,G,H,K,L)**. Immunohistochemistry; polyclonal rabbit anti- influenza A FPV/Rostock/34-virus-nucleoprotein antiserum; avidin-biotin-peroxidase-complex method; 3-amino-9-ethyl-carbazol chromogen (red-brown); hematoxylin counterstain (blue); Bars = 20 μm. More immunostaining slides are available upon request.

Distribution of influenza antigen in different organs was analyzed by IHC (Figure 6; Supplementary Table S3), and in lung and spleen samples using RT-qPCR (Supplementary Figure S3). Breed has a major effect on distribution of A/H5N1 in different organs. No NP-antigen was detected in Pekin ducks at 3 dpi using IHC, however, using RT-qPCR, 2.2.1.1 and 2.2.1.2 viruses were detected in the lungs, but not spleen, of Pekin ducks (Supplementary Figure S3). In contrast, in primary inoculated Muscovy ducks NP antigen was detected in neuroglial cells, cardiomyocytes, and thymocytes (Figure 6) as well as in a contact bird (data not shown). Variation in the distribution of 2.2.1.1 and 2.2.1.2 A/H5N1 antigen in Muscovy ducks was also observed. In Group 3, the antigen was not detected in the tracheal epithelium, liver, and kidneys; conversely all three ducks in group 4 had remarkable infiltration in these organs. In the gastrointestinal tract (duodenum, jejunum, cecal tonsils, proventriculus, and gizzard), the virus was detected only in birds of group 4, particularly in the neurons in peripheral ganglia. In Group 3, infected areas found in the lung and brain were less frequent compared to birds in group 4. In the lung of animals from group 4, virus antigen was detected in the upper and lower respiratory tract, while birds in group 3 showed viral antigen only in the bronchial epithelium. Viral antigen was not detected in endothelial cells in any organ or the circulatory system in Pekin or Muscovy ducks. Using RT-qPCR, viral RNA was also detected in the lungs and spleen of Muscovy ducks in groups 3 and 4 (Supplementary Figure S3).

Serum samples collected before infection were negative for AIV NP antibodies using ELISA. At the end of the experiment, sera from group 1 (*n* = 11), group 2 (*n* = 12), group 3 (*n* = 1), and group 4 (*n* = 2) were examined by NP-ELISA and HI test against homologous and heterologous antigens. Using ELISA, all sera post-infection were positive. Using HI test against 2.2.1.2-D antigen, all samples in groups 2 and 4 were positive (HI titer ≥8) with a mean titer of between 3.8 and 5 log_2_, in inoculated birds and 3.2 and 5 log_2_ in contact birds, respectively. Surviving birds in Group 1 and Group 3 were tested negative (HI titer < 8). Using 2.2.1.1 antigen, the sera of inoculated birds in groups 1 and 3 reacted at similar levels with a mean titer 4.7 and 6 log_2_, respectively, and no cross reaction with the 2.2.1.2-D antigen was obtained.

## Discussion

Since the introduction of 2.2.1 clade of A/H5N1 into poultry in Egypt in 2006, the virus established an endemic status and spilled over to humans making Egypt the country with the longest endemic status outside Asia and with the highest number of human infections. Understanding the factors driving the evolution and persistence of the Egyptian A/H5N1 may enable prediction and control of future outbreaks.

In this study, climatic factors during moderate to relatively cold months (October to March) where the number of outbreaks (i) is high and (ii) allows statistical analysis were collected. In summer, the prevalence of outbreaks is very low (Arafa A. et al., 2012; Arafa A. S. et al., 2012; El-Zoghby et al., 2013) and therefore statistical analysis will be misleading. Statistical analysis of selected climatic factors in this study indicated that ambient temperature influenced the prevalence of A/H5N1 outbreaks in Egypt from 2006 to 2015. Also, the thermostability of viruses from clade 2.2.1.1 and recent clade 2.2.1.2 at 56°C degrees is remarkable. Thus, we assume that the ability of some A/H5N1 viruses to survive at elevated temperatures (i.e., in summer season when temperature is over 40°C) is an important factor for the persistence and spread of H5N1 in Egypt. In a previous study, clade 2.2.1.2 virus was isolated from domestic ducks in mid-summer in Egypt (Hassan et al., 2013). Two major waves of the A/H5N1 in Egypt were reported due to the emergence of clade 2.2.1.1 virus in vaccinated poultry in 2008-2010 and clade 2.2.1.2 virus from October 2014 to March 2015. Viruses isolated from the two waves (2.2.1.1 and 2.2.1.2-B/2.2.1.2-C, respectively) exhibited increased thermal stability than the other viruses, which may be advantageous for virus persistence in harsh environment and spread when the average temperature is moderate (i.e., in winter months). Our results are partially in accordance with the analysis conducted by Murray and Morse (2011) who found that the incidence of A/H5N1 infections in humans in Egypt in 2006-2008 was strongly associated with moderate temperature and humidity. The current study does not explain the disappearance of clade 2.2.1.1 which was probably due to the extensive vaccination using local field-strains, competition with co-circulating H9N2 viruses and/or other unknown reasons (Abdelwhab et al., 2016; Hassan et al., 2016; Naguib et al., 2017). Moreover, climatic factors alone did not explain the high prevalence of HPAIV in domestic poultry in 2014/2015. Williams et al. (2011) suggested that anthropogenic factors (human population density, movement, etc.) were important for the spread of A/H5N1 in the Middle East and Northeastern Africa (Williams and Peterson, 2009). Likewise, increased incidence of A/H5N1 outbreaks in the commercial farms in Egypt was most strongly correlated with road network distances (Young et al., 2017).

All viruses replicated well and at similar levels in avian cell culture and had binding affinity to both avian and human-like receptors, meanwhile cell-to-cell spread was mostly virus-specific. An interesting observation is that the virus in clade 2.2.1.1, which is highly adapted to (vaccinated) chickens, replicated at lower levels in human lung cells than human-like viruses in clade 2.2.1.2. Mutations in clade 2.2.1.2 viruses increased replication in human cells while maintaining the ability for replication in avian cells (Watanabe et al., 2011b). Although this topic should be further investigated using a broader panel of clade 2.2.1.1 viruses, it has been postulated that the adaptation of A/H5N1 to terrestrial poultry may prevent the evolution of human-adapted viruses (Long et al., 2015). This may partially explain the lower prevalence of clade 2.2.1.1 viruses in humans compared to viruses in clade 2.2.1.2.

Ducks exhibit mild or no clinical signs after infection with A/H5N1 and, therefore, play an important role in the genesis and silent transmission of highly pathogenic viruses to susceptible gallinaceous poultry and probably to humans (Fan et al., 2014; Lee et al., 2014). However, the pathogenicity of H5N1 viruses in ducks may vary according to the duck species, age of ducks, virus strain and inoculation route (Pantin-Jackwood and Swayne, 2009; Szeredi et al., 2010; Cagle et al., 2011; Pantin-Jackwood et al., 2012, 2013; Yuan et al., 2014). In the recent 2014/2015 upsurge in poultry and humans in Egypt, ducks were speculated to be a major source for infection (Arafa et al., 2015). In this study, Muscovy ducks proved to be more sensitive than Pekin ducks which is in accordance with previous studies (Guionie et al., 2010; Cagle et al., 2011). Nearly all inoculated and in-contact Muscovy ducks died after infection with human-like clade 2.2.1.2 virus. Conversely, none of the Pekin ducks showed clinical signs or mortality, while producing antibodies and excreting a considerable amount of virus from the respiratory and digestive tract for up to 14 dpi. Viral RNA was detected in the lungs at 3 dpi. The virus was also transmitted to sentinel Pekin ducks without affecting health as shown by virus excretion in swabs and seroconversion. Previously, two Egyptian viruses from 2007 and 2008 from clade 2.2.1 and early clade 2.2.1.1 killed all 2-week-old Muscovy ducks, while 2008-virus killed all 2-week-old Pekin ducks and 2007-virus killed only 10 to 30% depending on the route of inoculation (Pantin-Jackwood et al., 2013). Furthermore, all Pekin ducks infected with Turkish A/H5N1 of clade 2.2 at 8-weeks-old died, while 12-week-old Pekin ducks survived the challenge without significant impact on the amount of virus excreted in both groups (Londt et al., 2008, 2010). Pekin and Muscovy ducks challenged intranasally with a clade 2.3.4 A/H5N1 died, after developing neurological signs, within 3.6 and 3.1 days, respectively (Cagle et al., 2011). Variable pathogenicity in domestic ducks may be due to variation in the immune response between the two breeds (Cagle et al., 2011). It is worth mentioning that virus excretion from “silently” infected ducks in this study is in accordance with intermittent excretion of Chinese A/H5N1 from clinically healthy ducks for up to 17 dpi (Hulse-Post et al., 2005). Together, the silent infection of Pekin ducks particularly with human-like clade 2.2.1.2 virus poses public health hazards and intervention strategies (e.g., targeted surveillance in Pekin ducks, segregation of Pekin ducks from backyards, etc.) should be considered.

### Limitations of the study

Some limitations for the current study should be considered: statistical analysis was conducted for a limited number of regions only, using national surveillance data (i.e., reported outbreaks) and a limited number of factors analyzed herein. Our model reveals an association between temperature and spread of A/H5N1. However, other factors can not be excluded. Moreover, we collected data for the outbreaks from official reports for national surveillance conducted by the ministry of agriculture in cooperation with the FAO. However, these numbers likely do not represent all cases observed in the field. Underreporting of outbreaks in Egypt is not uncommon due to lack of compensation for culling of infected flocks, false information on protection of poultry by vaccination, and masking infection (silent infection) due to partial protection induced by the vaccines (Vergne et al., 2012). Also, although governorates in this study are well known for their high-density poultry population, we did not find accurate estimates for annual poultry density in these four regions which may affect the number of outbreaks. Importantly, climatic weather for governorates in the Nile delta were not fully available. Lastly, we analyzed only a limited number of factors, while other potential important parameters such as elevation (Loth et al., 2011), chicken density (Pfeiffer et al., 2007), species of birds and density of human population (Gilbert et al., 2008b), and movement of birds, particularly for marketing or hatcheries to rearing areas should be included in future models. Likewise, the impact of seasonal variation on movement or migration of feral or wild birds should be also considered. Biological fitness was assessed using representative viruses from each clade; however, more viruses particularly from clade 2.2.1.1 should be analyzed in the future. Clinical outcome in inoculated ducks with A/H5N1 may be affected by viral doses and ages of ducks (Londt et al., 2010; Pantin-Jackwood et al., 2012) which should be considered in the future. In summary, our study sets a baseline on the importance of several parameters in shaping the epidemiological situation of A/H5N1 infection in poultry and humans in Egypt.

## Author contributions

EA, TM, JV, and HH conceived and designed the study. AS, DS, and EA conducted the animal experiment. EK, HE, and EA collected data and conducted the climatic factors analysis. MH, AA, WH, and HA provided the official data on the outbreaks in Egypt and/or viruses used in this study. RU conducted the histopathological analysis. EK and EA conducted the statistical analysis. AS, DS, MG, and EA conducted the *in-vitro* characterization. EA wrote the manuscript. All authors read and approved the final manuscript.

### Conflict of interest statement

The authors declare that the research was conducted in the absence of any commercial or financial relationships that could be construed as a potential conflict of interest.

## Abbreviations

A/H5N1: Highly pathogenic avian influenza virus H5N1
A549: Adenocarcinomic human alveolar basal epithelial cells
AIC: Akaike information criterion
AIV: Avian influenza viruses
BSA: Bovine serum albumin
CBS: Citrate-buffered saline
CEK: Chicken embryo kidney cells
dpi: days post-inoculation
ECE: Embryonated chicken eggs
FLI: Friedrich-Loeffler-Institut
HA test: hemagglutination test
HA: hemagglutinin
HPAIV: Highly pathogenic avian influenza virus
MDCKII: Madin-Darby canine kidney cells II
NA: Neuraminidase
NLQP: National Laboratory for Veterinary Quality Control on Poultry Production
PFU: Plaque-forming unit
SA: Sialic acid
SPF: Specific pathogen free
TRBCs: Turkey erythrocytes.
