# Supplementary material for: Potential Biological and Climatic Factors That Influence the Incidence and Persistence of Highly Pathogenic H5N1 Avian Influenza Virus in Egypt
Source: Front Microbiol. 2018 Mar 27;9:528. doi: 10.3389/fmicb.2018.00528 (PMC5880882; doi:10.3389/fmicb.2018.00528)
Supplement: Supplementary file 1 [file Table1.PDF]

Supplementary Table S1: Negative binomial regression models for explaining the observed number of A/H5N1 outbreaks in winter seasons for the nation-level data (i.e. all Egypt, d.f. – degrees of freedom).

	**Model parameters**				**Model performance**				
		β	SE	P-value (Wald test)	Deviance	P-value (χ^2 test, goodness of fit)	AIC	2 x log-likelihood	P-value (Likelihood ratio test to full model)
**Model 1 (full model)**	Temp (min)	-2.46	1.36	0.07	9.08 (3 d.f.)	0.03	100.62	-86.62	-
	Temp (max)	-2.60	0.40	< 0.01					
	Temp (average)	5.46	1.19	< 0.01					
	Humidity	0.22	0.08	< 0.01					
	Wind	0.72	0.14	< 0.01					
**Model 2**	Temp (max)	-1.50	0.62	0.02	9.24 (6 d.f.)	0.16	109.08	-101.08	< 0.01
	Temp (average)	1.79	0.69	< 0.01					
**Model 3 (null model)**					9.47 (8 d.f.)	0.30	112.00	-108.00	< 0.01
