# Supplementary material for: Potential Biological and Climatic Factors That Influence the Incidence and Persistence of Highly Pathogenic H5N1 Avian Influenza Virus in Egypt
Source: Front Microbiol. 2018 Mar 27;9:528. doi: 10.3389/fmicb.2018.00528 (PMC5880882; doi:10.3389/fmicb.2018.00528)
Supplement: Supplementary file 2 [file Table2.PDF]

Supplementary Table S2: Virus excretion in ducks inoculated with two different Egyptian A/H5N1 viruses

Dpi	Swabs	Ducks	Pekin ducks		Muscovy ducks	
			Group 1 H5N1/2.2.1.1	Group 2 H5N1/2.2.1.2	Group 3 H5N1/2.2.1.1	Group 4 H5N1/2.2.1.2
Day 2	Oral	inoculated	1.0±0.3 (6/10)	2.4±1.2 (10/10)	4.0±0.7 (10/10)	5.0±1.5 (10/10)
		contact	0.9±0.3 (3/5)	2.7±0.9 (5/5)	2.4±1.7 (5/5)	3.9±1.7 (5/5)
	Cloacal	inoculated	1.6±1 (8/10)	2.3±0.7 (10/10)	1.3±0.7 (10/10)	3.4±0.9 (10/10)
		contact	1.9±1.5 (4/5)	2.5±0.9 (5/5)	1.0±0.9 (4/5)	1.6±1.5 (5/5)
Day 4	Oral	inoculated	1.5±0.9 (6/7)	2.8±1.5 (7/7)	5.0±0.3(7/7)	5.7±0.6 (6/6)
		contact	1.9±0 (1/5)	2.1±0.5 (5/5)	4.7±0.4(5/5)	6.1±0.7 (4/4)
	Cloacal	inoculated	1.2±0.8 (5/7)	1.7±1.2 (6/7)	3.0±1.2 (7/7)	4.4±0.5 (6/6)
		contact	2.2±1.0 (4/5)	1.5±0.6 (5/5)	2.7±1.2 (5/5)	6.1±0.7 (4/4)
Day 7	Oral	inoculated	0.7±1.8 (4/7)	1.0±0.5 (2/7)	3.0±0.4 (3/3)	3.0 (1/1)
		contact	0 (0/5)	2.7±1.4 (4/5)	4.5±0.9 (3/3)	4.2±1.3 (2/2)
	Cloacal	inoculated	2.3±0.6 (5/7)	2.1±0.3 (3/7)	1.9±0.7 (2/3)	2.6 (1/1)
		contact	1.0±1.1 (2/5)	1.4±1.0 (4/5)	3.3±1.4 (3/3)	2.6±0.2 (2/2)
Day 11	Oral	inoculated	0.8±0.5 (4/6)	0.1 (1/7)	1 (1/1)	0.2 (1/1)
		contact	0.5±0.5 (3/5)	0 (0/5)	dead	0.9 (1/1)
	Cloacal	inoculated	0.6 (1/6)	1.1±0.9 (3/7)	0	0
		contact	0.3 (1/5)	1.1±0.3 (3/5)	dead	0.6 (1/1)
Day 14	Oral	inoculated	0.7 (1/6)	1.3±0.8 (2/7)	0	0.6 (1/1)
		contact	0	1.6±0.4 (2/5)	dead	0
	Cloacal	inoculated	0	1.3±0.1 (2/7)	0	0
		contact	0.9± (3/5)	1.0±0.3 (4/5)	dead	0

n.a.= not applicable since all birds died

Shown is the average of virus excretion expressed as log10 PFU/ml ± standard deviation. Given in parentheses is the number of positive birds/number of total examined at each time point.
