# Supplementary material for: Potential Biological and Climatic Factors That Influence the Incidence and Persistence of Highly Pathogenic H5N1 Avian Influenza Virus in Egypt
Source: Front Microbiol. 2018 Mar 27;9:528. doi: 10.3389/fmicb.2018.00528 (PMC5880882; doi:10.3389/fmicb.2018.00528)
Supplement: Supplementary file 3 [file Table3.PDF]

Supplementary Table S3: Tissue distribution of nucleoprotein of H5N1 viruses in Pekin (Groups 1 and 2) and Muscovy (Groups 3 and 4) ducks at day 3 post inoculation using immunohistochemistry

		Lung			Trachea	Nervous system			Heart	Pancreas	Liver		Kidney	Thymus
	Bird	bronchial epithelium	parabronchial epithelium	pneumocytes	epithelium	neurons, glial cells	ependymal cells	meningeal cells	myocytes	acinar cells	hepatocytes	Kupffer cells	tubular epithelium	
Gr1	1	0	0	0	0	0	0	0	0	0	0	0	0	0
	2	0	0	0	0	0	0	0	0	0	0	0	0	0
	3	0	0	0	0	0	0	0	0	0	0	0	0	0
Gr2	1	0	0	0	0	0	0	0	0	0	0	0	0	0
	2	0	0	0	0	0	0	0	0	0	0	0	0	0
	3	0	0	0	0	0	0	0	0	0	0	0	0	0
Gr3	1	1	0	0	0	1	0	0	2	0	0	0	0	2
	2	0	0	0	0	3	3	0	3	2	0	0	0	2
	3	2	0	0	0	2	0	0	2	1	0	0	0	2
Gr4	1	3	3	3	2	4	3	2	3	3	3	0	2	3
	2	2	0	1	2	3	2	0	3	3	2	0	0	4
	3	0	0	0	2	1	3	0	2	2	2	0	0	3

The intensity of signals of influenza nucleoprotein was semi-quantified by scoring on a 0 to 4 severity scale for tissues: 0 = negative; 1= single cells, 2= scattered foci, 3= numerous foci, 4= coalescing foci or diffuse and on a scale of 0 to 3 for endothelium: 0 = negative; 1= single blood vessel, 2 = multiple blood vessels, 3= diffuse
