# Supplementary material for: Potential Biological and Climatic Factors That Influence the Incidence and Persistence of Highly Pathogenic H5N1 Avian Influenza Virus in Egypt
Source: Front Microbiol. 2018 Mar 27;9:528. doi: 10.3389/fmicb.2018.00528 (PMC5880882; doi:10.3389/fmicb.2018.00528)

Supplementary Figure S1: Predicted number of A/H5N1 outbreaks from the so-called reduced model (blue line) with standard errors (blue shaded area). Black dots represent the actual observed number of A/H5N1 outbreaks in domestic poultry.

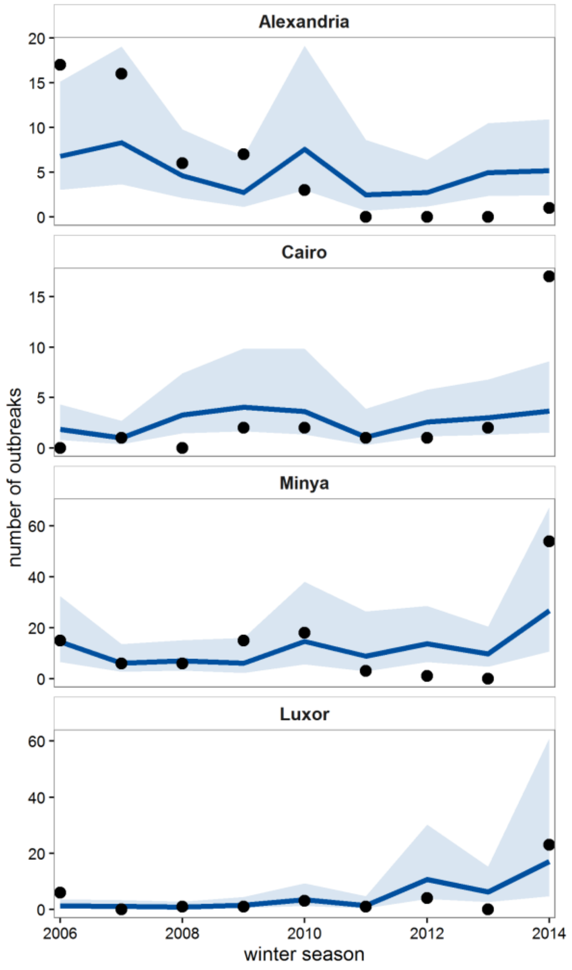

Supplement: Supplementary file 4 [file Image1.PDF]
