# Supplementary material for: Potential Biological and Climatic Factors That Influence the Incidence and Persistence of Highly Pathogenic H5N1 Avian Influenza Virus in Egypt
Source: Front Microbiol. 2018 Mar 27;9:528. doi: 10.3389/fmicb.2018.00528 (PMC5880882; doi:10.3389/fmicb.2018.00528)

Supplementary Figure S2: Predicted number of A/H5N1 outbreaks from the model based on average and maximum temperature per winter season for all Egypt (blue line) with standard errors (blue shaded area). Black dots represent the actual observed number of A/H5N1 outbreaks in domestic poultry.

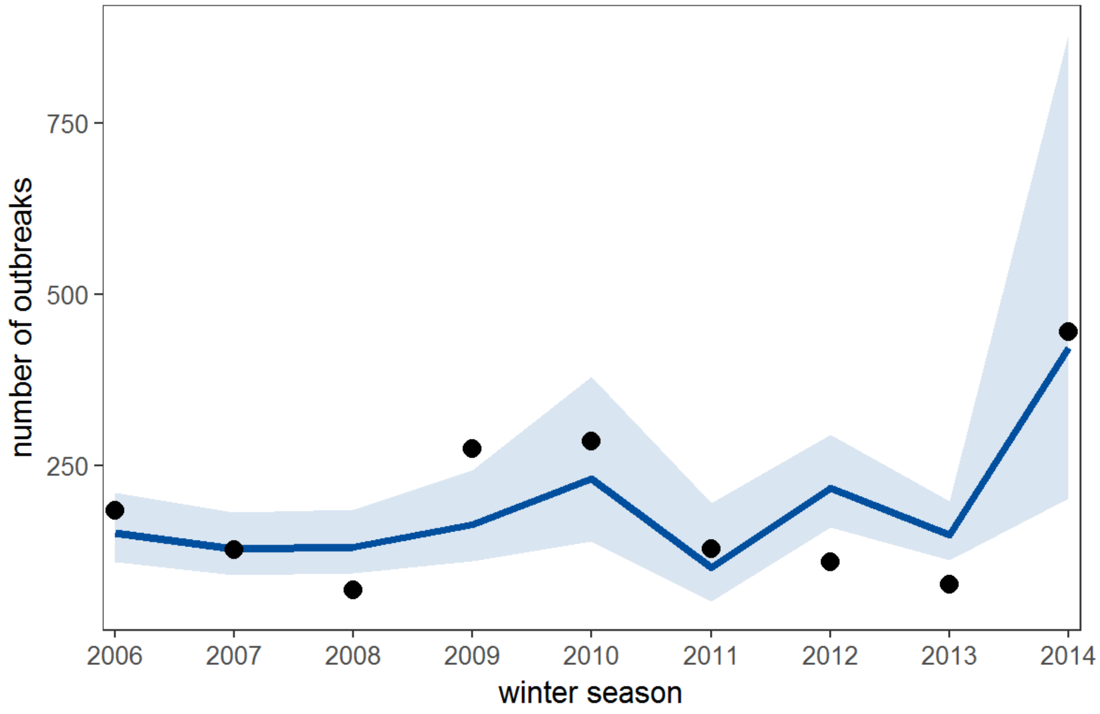

Supplement: Supplementary file 5 [file Image2.PDF]
