# Supplementary material for: Potential Biological and Climatic Factors That Influence the Incidence and Persistence of Highly Pathogenic H5N1 Avian Influenza Virus in Egypt
Source: Front Microbiol. 2018 Mar 27;9:528. doi: 10.3389/fmicb.2018.00528 (PMC5880882; doi:10.3389/fmicb.2018.00528)
Supplement: Supplementary file 6 [file Image3.PDF]

Supplementary Figure S3: Results of RT-qPCR for the detection of 2.2.1.1 and 2.2.1.2 in lungs and spleen of inoculated ducks at 3 days post inoculation

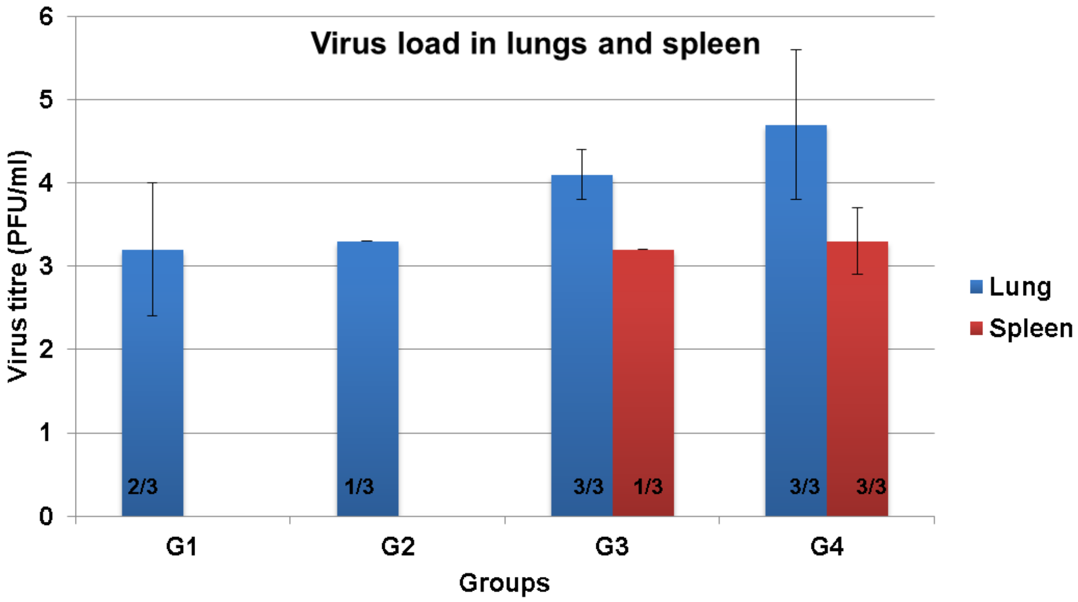

Number of positive birds out of 3 examined Pekin (G1 and G2) and Muscovy (G3 and G4) ducks at 3 dpi is shown. The virus was not detected from the spleen samples obtained from Pekin ducks.
